# Correlation Analysis of the Bacterial Community and Wood Properties of *Populus* × *euramericana* cv. “74/76” Wet Heartwood

**DOI:** 10.3389/fmicb.2022.868078

**Published:** 2022-07-04

**Authors:** Jianmin Fan, Shijie Wang, Changjun Ding, Changming Ma, Xinghao Chen, Jinmao Wang, Minsheng Yang, Xiaohua Su

**Affiliations:** ^1^Forest Department, Forestry College, Hebei Agricultural University, Baoding, China; ^2^State Key Laboratory of Tree Genetics and Breeding, Research Institute of Forestry, Chinese Academy of Forestry, Beijing, China; ^3^Hebei Key Laboratory for Tree Genetic Resources and Forest Protection, Baoding, China; ^4^Key Laboratory of Tree Breeding and Cultivation of State Forestry Administration, Research Institute of Forestry, Chinese Academy of Forestry, Beijing, China

**Keywords:** *Populus* × *euramericana* cv. “74/76”, wetwood, heartwood, high-throughput sequencing, bacterial diversity, bacterial community structure

## Abstract

Wetwood disease of poplar limits the processing and manufacturing of poplar, and the pathogenic bacteria of wet heartwood are poorly known. We used high-throughput sequencing methods to analyze the bacterial community of the heartwood, sapwood, root tissue, and rhizosphere soil of *Populus* × *euramericana* cv. “74/76” (poplar 107) in wetwood trees and healthy trees to explore the cause of poplar wetwood disease. Bacterial diversity and community structure were analyzed, and the correlation between wood properties and bacterial relative abundance was analyzed to explore their relationship. Two alpha-diversity indices of endophytic bacteria in the heartwood of wetwood trees were significantly (*p* < 0.05) lower than that in the heartwood of healthy trees, and the community structure between the two types of trees was significantly different. No significant differences in the alpha-diversity indices nor community structure were observed in the sapwood, root tissue, or rhizosphere bacterial community of diseased and healthy trees. The distribution of dominant bacteria genus in the heartwood of diseased and healthy trees differed. *Proteiniphilum*, *Actinotalea*, and *Methanobacterium* were the dominant genera in diseased trees’ heartwood. *Proteiniphilum*, *Dysgonomonas*, and *Bacteroides* were the dominant genera in healthy trees’ heartwood. The relative abundance of *Proteiniphilum*, *Actinotalea*, and *Methanobacterium* was significantly higher in the heartwood of wetwood trees than those of healthy trees. A db-RDA analysis found that these three bacterial genera were positively correlated with the rate of wet heartwood. These three bacterial genera may be the main pathogens causing poplar wetwood disease.

## Introduction

Wet heartwood, also called wetwood disease, is an abnormal tissue formed in living standing trees’ heartwood. Its typical characteristics are higher water content than healthy heartwood, a darker tissue color, and a stench of fatty acids. Wetwood is a common tree disease that occurs in coniferous and broad-leaved trees (Alizadeh et al., 2017a,b; Basavand et al., 2021; Martin et al., 2021). The wetwood disease has been studied since the 20th century in species such as white fir (Wilcox and Pong, 1971; Wilcox and Oldham, 1972), *Abies lasiocarpa* [Hook] Nutt (Zhang et al., 2006), *Pinus sylveatris* L. (Kovaleva et al., 2015), *Abies balsamea* (L.) Mill. (Jeremic et al., 2004), *Abies alba* Mill. (Streichan, 1986; Martin et al., 2021), and other coniferous species, as well as *P. deltoides* cv. *Lux ex*. I-69/55 (Yu et al., 2019), *Betula pendula* roth (Goychuk et al., 2020), *Ulmus* spp. L. (Alizadeh et al., 2017a,b), *Morus* (Basavand et al., 2021), *Casuarina equisetifolia* (Ayin et al., 2019), *Gmelina arboreaI* (Moya et al., 2009), and other broad-leaved tree species. Many things have been investigated to identify the causes for development of wetwood, such as genetic variability, bacterial and fungal communities, wood physicochemical properties, and wood processing and utilization (Schroeder and Kozlik, 1972; Schink et al., 1981a; Xu et al., 2001; Jeremic et al., 2004; Zhang et al., 2005). Wetwood is mainly caused by a bacterial infection, related to physical, mechanical, and biological damage (Moya et al., 2009; Alizadeh et al., 2017a; Martin et al., 2021). Some researchers have reported that pathogens responsible for the development of wetwood are endophytic bacteria of the tree. And they thought that bacteria were part of the natural microbes of the normal wood, generally in a dormant state, and only became active when the tree species changed in a way that would provide nutrients for its inhabitants, leading to the development of wetwood (Zu, 2000). Wetwood has been researched extensively but its cause and underlying process of formation remain unclear (Wang et al., 2008). Wetwood disease is an important problem for the forest and lumber products industry because it often takes longer to dry and may crack, yielding a lumber with diminished quality (Jeremic et al., 2004; Roque et al., 2009). Furthermore, wet heartwood may deteriorate glue bond strength, lowering the quality of plywood and reconstituted wood products (Xu et al., 2010).

One study compared the anatomical structure of wet and healthy heartwood in *A. balsamea* (L.) Mill., and reported no significant differences in their characteristics except for the higher frequency of bacteria (Jeremic et al., 2004). A variety of pathogenic bacteria have been isolated from wet heartwood tissues. Strains of Enterobacteriaceae are most often isolated from diseased trees, particularly *Erwinia* and *Klebsiella*, as well as *Enterobacter*, *Pantoea*, *Citrobacter*, *Edwardsiella*, *Lactobacillus*, and *Kosakonia* (Carter, 1945; Hartley et al., 1961; Knutson, 1973; Schink et al., 1981b; Murdoch, 1983; Diamandis and Koukos, 1992; Zheng et al., 2006; Ayin et al., 2019; Goychuk et al., 2020). Other bacteria are also often separated from the wet heartwood of conifers and broadleaf trees, such as *Bacillus*, *Clostridium*, *Bacteroides*, *Ralstonia*, *Xanthomonas*, and *Pseudomonas* (Carter, 1945; Knutson, 1973; Schink et al., 1981b; Murdoch, 1983; Warshaw et al., 1985; Diamandis and Koukos, 1992; Kovaleva et al., 2015; Ayin et al., 2019; Goychuk et al., 2020).

The symptoms of wet heartwood occur in many tree species, including *Leuce*, *Aigeiros*, *Tacamahaca*, and *Populus* (Song et al., 2017). Poplar is one of the most important species for short-cycle industrial raw materials and for constructing ecologically protected forests in China (Song et al., 2017). Wet heartwood is prone to cracking, warping, shrinkage, and other defects during the drying process, which reduces the value of the wood (Jeremic et al., 2004; Roque et al., 2009). Traditional bacterial culture methods do not fully reflect the composition and diversity of the bacterial communities of wetwood trees, which limits the study of the disease. In this study, *Populus* × *euramericana* cv. “74/76” (poplar 107) from 25 plantations were used as research materials. Wood properties of diseased and healthy trees were analyzed and compared to explore the effect of wetwood disease on wood properties. Additionally, the composition and diversity of the bacterial community in the heartwood, sapwood, root and rhizosphere soil of the wetwood and healthy trees were evaluated by high-throughput sequencing. The bacteria possibly involved in wetwood formation were analyzed by comparing bacterial communities between different locations of diseased and healthy trees. The results have important implications for further understanding the development of wetwood disease and also provide new insights into the role of bacterial communities in plant health.

## Materials and Methods

### Sample Collection

**Figure 1:**
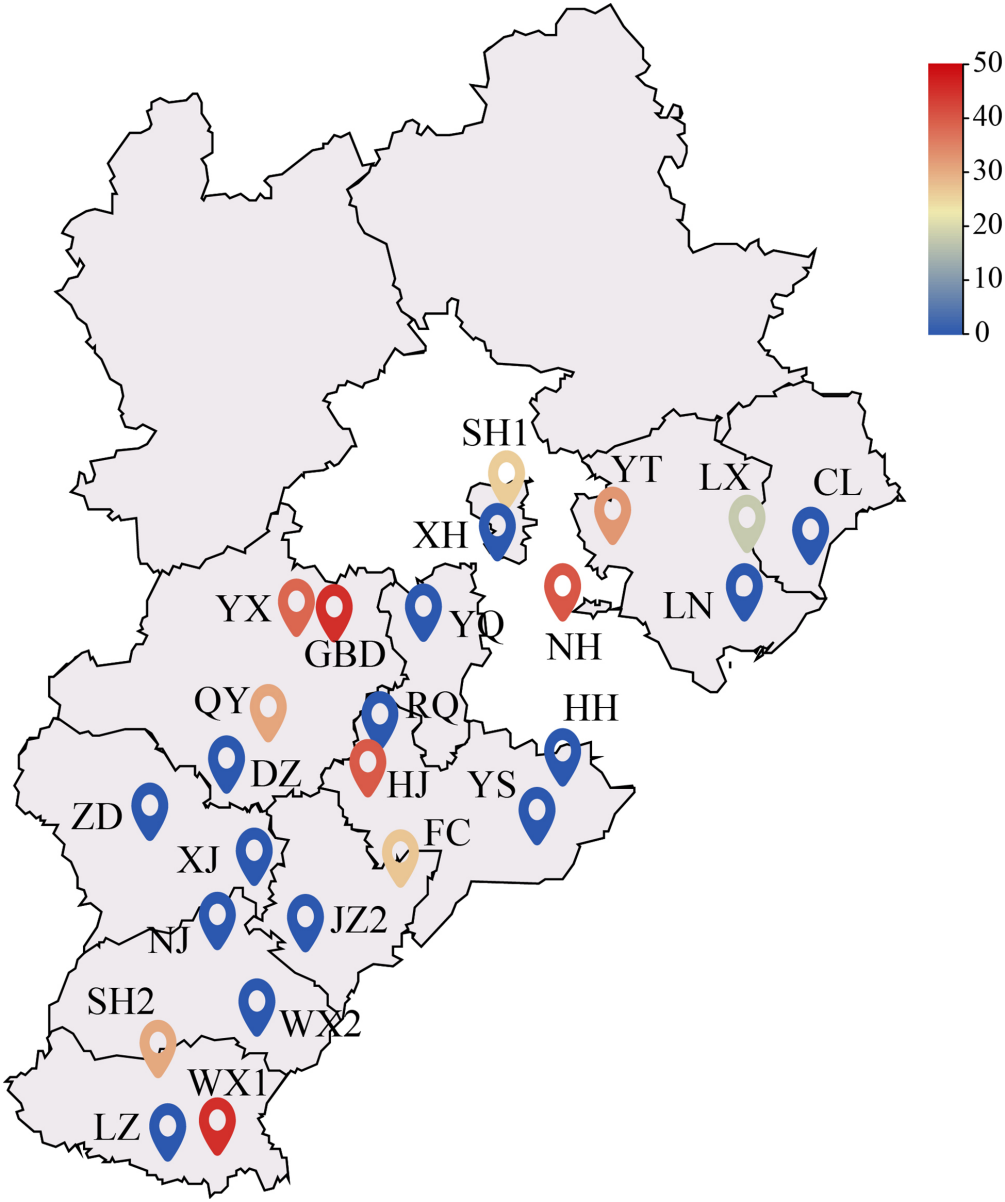
Distribution of the sampling sites. The color of the sampling point icon indicates the size of the wet heartwood rate at that site. The smaller the wet heartwood percentage, the bluer the color of the icon, the higher the wet heartwood percentage, and the redder the color of the icon.

**Figure 2:**
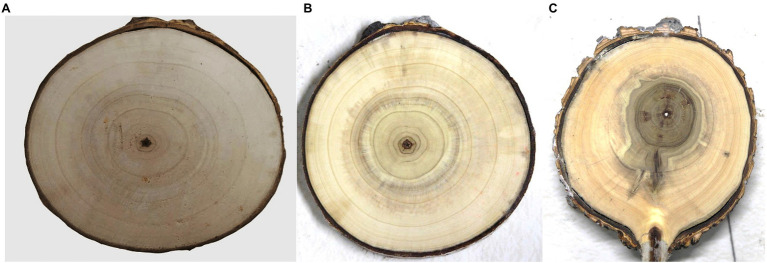
The disease condition of wet heartwood disease in different degrees. **(A)** Healthy heartwood; **(B)** Wet heartwood with mild symptoms; and **(C)** Wet heartwood with more severe symptoms.

In July 2017, a random sampling method was used to investigate 25 poplar plantation in Hebei Plain, China. One standard tree was selected from each of the 25 plantations wood property testing. The plantation trees were 5–9 years of age and were not maintained. The forest density was 802 trees/h·m^−2^. The location of each sample is shown in Figure 1, and the basic information of the sample wood is shown in Table 1. After the standard wood was felled, the wet heartwood area was immediately visible. About 11 of the 25 trees were diseased trees, and the other 14 were healthy trees (Figure 2). A 5 cm thick disk was sawed at the base of the trunk (0 m above ground) to determine the color difference and the wet heartwood ratio of the wood. Two 5 cm thick disks were sawed at breast height (1.3 m above ground). One disk was used to determine the chemical properties of the wood, and the other disk was stored at −80°C to determine the bacterial community composition. A 1 m long peeling block was sawed at 2–3 and at 3–4 m from the base of each tree to determine physical and mechanical properties of the wood. Rhizosphere soil samples were collected by the shaking soil method (Hua et al., 2012). The samples were taken 40 cm from the base of the target plant at each cardinal point (east, west, south, and north) and at a soil depth of 20 cm. The nonrhizosphere soil without the roots was shaken off, and sterile tweezers were used to gently peel off the soil attached to the surface of the root system. After the rhizosphere soil was obtained, filled a 10 ml centrifuge tube with the soil by the quarter method (Bao, 2000), and stored at −80°C prior to DNA extraction. Root and soil samples were taken at the same sampling point. The tender roots were taken from the root tips, placed in a 10 ml centrifuge tube, and stored at −80°C prior to DNA extraction.

**Table 1:** Sample information and wet heartwood rate.

Sampling Point	Number	Tree age (year)	Heartwood type	Wet heartwood rate (%)
Changli County	CL	9	Healthy	0
Dingzhou City	DZ	5	Healthy	0
Fucheng County	FC	9	Diseased	26.95
Gaobeidian City	GBD	6	Diseased	45.28
Huanghua City	HH	7	Healthy	0
Hejian City	HJ	7	Diseased	40.09
Jizhou Qu	JZ2	8	Healthy	0
Luannan County	LN	7	Healthy	0
Luan County	LX	7	Diseased	17.84
Linzhang County	LZ	9	Healthy	0
Ninghe County	NH	7	Diseased	40.44
Ningjin County	NJ	8	Healthy	0
Qingyuan County	QY	6	Diseased	30.97
Renqiu City	RQ	8	Healthy	0
Sanhe City	SH1	6	Diseased	26.14
Shahe City	SH2	9	Diseased	30.46
Wei County	WX1	7	Diseased	45.33
Weixian County	WX2	6	Healthy	0
Xianghe County	XH	8	Healthy	0
Xinji City	XJ	8	Healthy	0
Yongqing County	YQ	7	Healthy	0
Yanshan County	YS	6	Healthy	0
Yutian County	YT	7	Diseased	32.69
Yi County	YX	7	Diseased	38.48
Zhengding County	ZD	7	Healthy	0

*The wet heartwood rate of the healthy trees was recorded as 0%*.

### Determining the Color Difference and Wet Heartwood Rate of the Wood

The surface of the wood disk was scraped after natural air drying. According to the standard colorimetric theory of the International Commission on Illumination (CIE) Lab (Li et al., 2018), the chroma values L^*^, a^*^, and b^*^ of the heartwood were measured with an automatic colorimeter, and each disk was measured three times.

A ruler was used to measure the diameter of the disk (with the bark removed) and the diameter of the wet heartwood part of the disk. The diameter of the wet heartwood of the healthy trees was recorded as 0 mm. Each disk was measured twice, and the average value was taken to obtain the total radius *R*_1_ and the wet heartwood radius *R*_2_ of the disk. The wet heartwood rate was calculated according to the following formula:


$$\mathrm{Wet}\;\mathrm{heartwood rate}=\frac{R_{2}^{2}}{R_{1}^{2}}\times 100\%$$

### Determining the Chemical, Physical, and Mechanical Properties of the Wood

A disk was cut at breast height and the heartwood was separated from the sapwood. The test material was air-dried, cut into small slices, crushed with a pulveriser, and passed through a 60-mesh sieve prior to analyses. The contents of cellulose, hemicellulose, and lignin were measured in the heartwood and sapwood using the method described by Yin et al. (2017). The contents of cold/hot water and 1% NaOH extractive were measured following the guidelines GB/T 2677.4-1993 [Standardization Administration of China (SAC), 1993a] and GB/T 2677.5-1993 [Standardization Administration of China (SAC), 1993b], respectively. Each sample was measured three times, and the average value was taken.

The wood density and shrinkage rate of the heartwood and sapwood were measured in accordance with GB/T 1933-2009 [Standardization Administration of China (SAC), 2009a] and GB/T 1932-2009 [Standardization Administration of China (SAC), 2009b], respectively. Bending strength, the bending elastic modulus, and compressive strength parallel to the grain of the heartwood and sapwood were measured in accordance with GB/T 1936-1-2009 [Standardization Administration of China (SAC), 2009c], GB/T 1936-2-2009 [Standardization Administration of China (SAC), 2009d], and GB/T 1935-2009 [Standardization Administration of China (SAC), 2009e], respectively.

### DNA Extraction and High-Throughput Sequencing

DNA was extracted from the heartwood (H), sapwood (S), root (R), and rhizosphere soil (SP) samples using a modified CTAB method (Porebski et al., 1997). The 341F (5′-CCTAYGGGRBGCASCAG-3′) and 806R (5′-GGACTACNNG GGTATCTAAT-3′) primers were used for PCR amplification of 16S rRNA. After successful amplification, the PCR products were purified by the AxyPrep DNA Gel Extraction Kit (Axygen Biosciences, Union City, CA, United States) according to the manufacturer’s instructions and quantified using ABI StepOnePlus Real-Time PCR System (Life Technologies, Foster City, United States). Purified amplicons were pooled in equimolar amounts and paired-end sequenced (2 × 250 bp) on an Illumina MiSeq platform according to the standard protocols. The Illumina data generated in this study were deposited in the NCBI Sequence Read Archive and are available under the project number PRJNA832960.

### Bioinformatics and Statistical Analysis

After the raw reads were obtained by sequencing, the low-quality reads were filtered using FASTP software, and the paired-end reads were spliced into raw tags using FLASH (v1.2.11) software. The raw tags were filtered using Qiime (v1.9.1) to obtain clean tags, and chimeras were removed based on the UCHIME algorithm in USEARCH software. The Uparse algorithm in Usearch software was used to cluster the effective tag sequences, and cluster them as OTUs with a similarity of 97% by default. Alpha-diversity indices were calculated using an iterative subsampling procedure based on the samples with the lowest reads counts (23,000 sequences) as implemented in QIIME in order to account for differences in read counts across samples. The differences in alpha-diversity indices between healthy trees and wetwood trees were analyzed using the Wilcoxon rank-sum test after checking normal distributions of data using the Shapiro–Wilk test and analyzing homoscedasticity of variances of data using the Bartlett’s test in the Vegan package of R language. The differences in bacterial relative abundance between healthy trees and wetwood trees were analyzed using the Wilcoxon rank-sum test in the Vegan package of R language. Non-metric multidimensional scaling (NMDS) analysis was performed using the Bray–Curtis distance. SPSS (v26.0) software was used to calculate Spearman correlation coefficient between microorganisms and the wood chroma values, wet heartwood rate, chemical properties, and physical and mechanical properties of the wood. The variables with a significant correlation were selected and the Cytoscape software was used to draw correlation network diagrams. A db-RDA analysis were executed in R project Vegan package (version 2.5.3) to clarify the influence of endophytic bacteria of heartwood and sapwood on wood variables of heartwood and sapwood. Parametric assumptions test and independent samples *t*-test were performed with SPSS (v26.0) software to analyze the differences in the chemical properties and physical and mechanical properties of the wood between the groups. Cluster analysis of the chroma value and wet heartwood rate of wood was performed using Past (v4.0) software.

## Results and Analysis

### Chroma Value and Wet Heartwood Rate of the Wood

The heartwood chroma values L^*^, a^*^, and b^*^ of 25 samples were measured using an automatic colorimeter. The L^*^ value (70.14) of the wetwood trees (WW) was lower than that of healthy trees (NW; 71.29), indicating that the heartwood of WW was less bright and darker in color. The a^*^ value (7.16) and b^*^ value (14.79) of the WW were greater than those of NW (6.91 and 14.64, respectively), indicating that the color of the heartwood of WW was reddish-brown (Supplementary Figure S1). The wet heartwood rate of the WW ranged from 17.84 to 45.34%, and it was significantly larger than NW (*p* < 0.05; Table 1). Cluster analysis of 25 samples based on the chroma value and the wet heartwood rate showed that the WW samples were clustered into one category except for FC, and the NW samples were clustered into another category (Supplementary Figure S2). Therefore, our subsequent analyses excluded the FC sample.

### Chemical, Physical, and Mechanical Properties of the Wood

**Figure 3:**
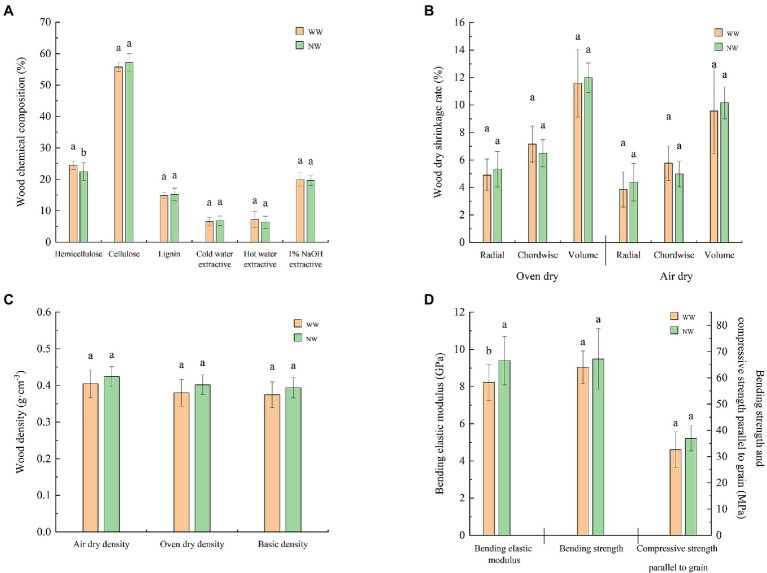
Chemical properties and physical and mechanical indicators of the heartwood. **(A)** Chemical composition of the heartwood; **(B)** Dry shrinkage rate of the heartwood; **(C)** Density of the heartwood; and **(D)** Mechanical indicators of the heartwood. The letter represents a significant difference at the *p* < 0.05 level. WW and NW represent diseased and healthy trees, respectively.

The hemicellulose content of wet heartwood was significantly (*p* < 0.05) higher than healthy heartwood (Figure 3A) and the bending elastic modulus of the heartwood was significantly (*p* < 0.05) lower than normal heartwood (Figure 3D). However, the other indicators of heartwood chemical composition (Figure 3A), heartwood dry shrinkage rate (Figure 3B), heartwood density (Figure 3C), and heartwood bending elastic modulus (Figure 3D) were not significantly different between healthy and diseased trees.

**Figure 4:**
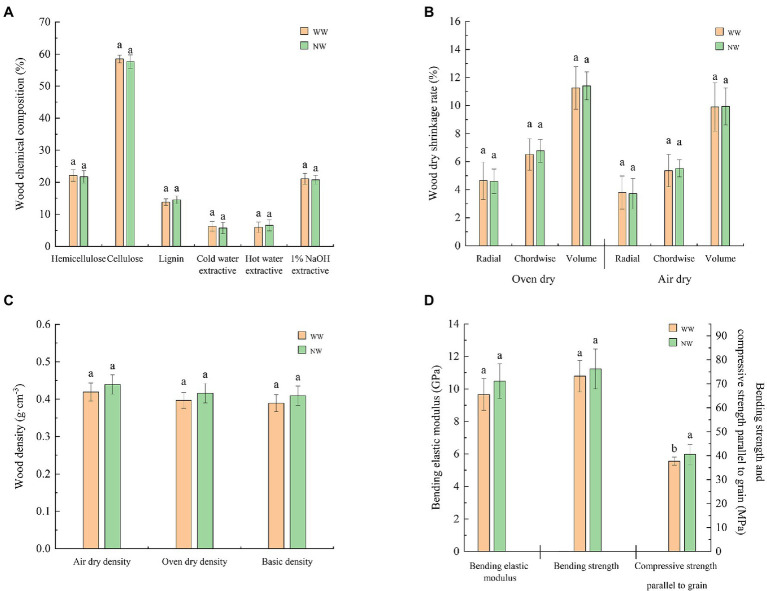
Chemical properties and physical and mechanical indicators of the sapwood. **(A)** Chemical composition of the sapwood; **(B)** Dry shrinkage rate of the sapwood; **(C)** Density of the sapwood; and **(D)** Mechanical indicators of the sapwood. The letter represents a significant difference at the *p* < 0.05 level. WW and NW represent diseased and healthy trees, respectively.

The compressive strength parallel to the grain of the sapwood in diseased trees was significantly (*p* < 0.05) lower than in healthy trees (Figure 4D). However, the other indicators of sapwood chemical composition (Figure 4A), sapwood dry shrinkage rate (Figure 4B), sapwood density (Figure 4C), and sapwood bending elastic modulus (Figure 4D) were not significantly different between healthy and diseased trees.

### Microbial Community Diversity

**Figure 5:**
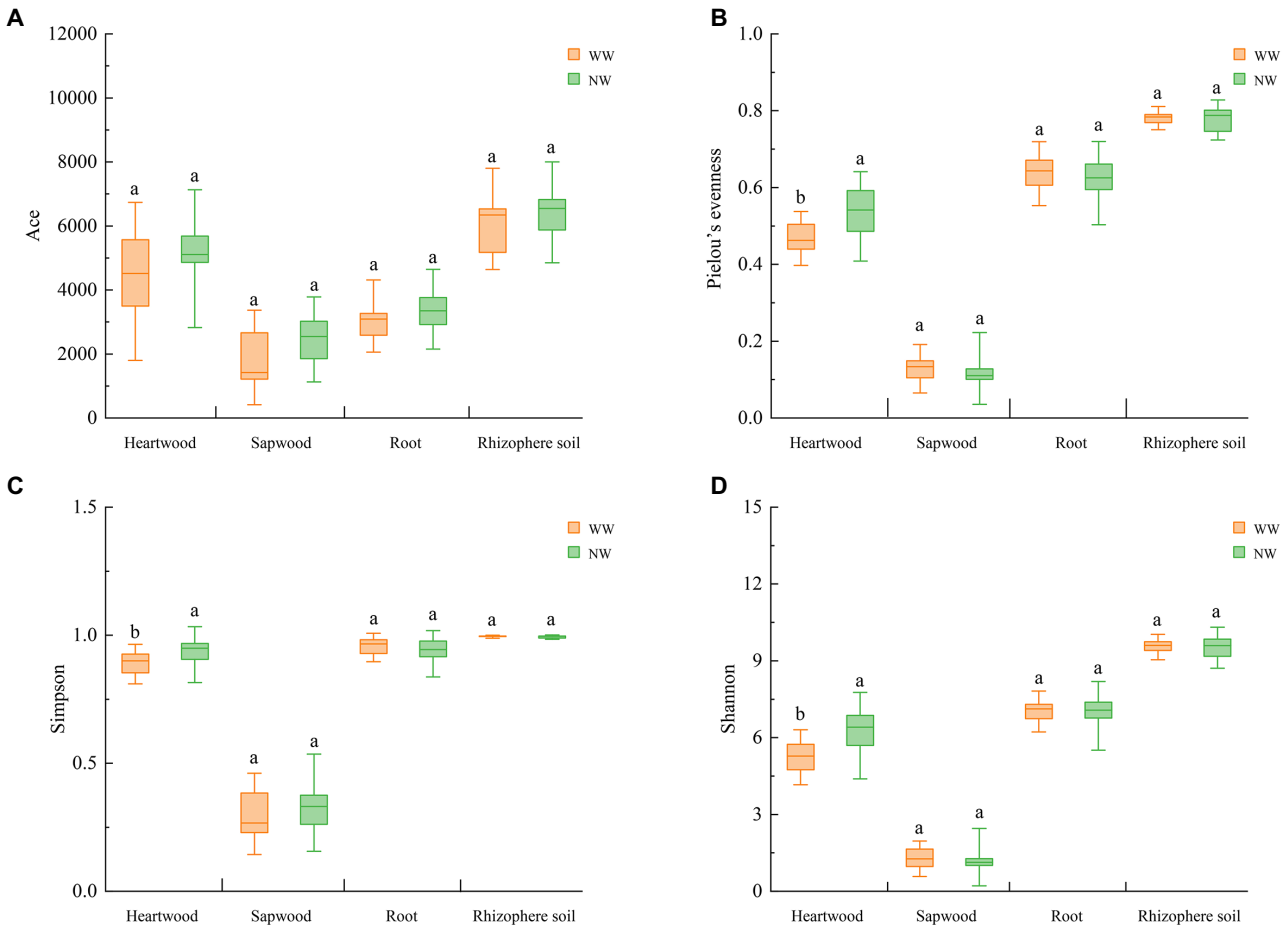
Alpha indices of the different samples. **(A)** Ace index; **(B)** Pielou’s evenness index; **(C)** Simpson index; and **(D)** Shannon index. The letter represents a significant difference at the *p* < 0.05 level. WW and NW represent diseased and healthy trees, respectively.

The number of effective tags ranged from 103,280 to 191,493 after strictly filtering the original data. The number of OTUs ranged from 1,034 to 4,987, and the good coverage rate ranged from 0.985 to 0.997. The number of WW OTUs at the different sampling locations was less than those of NW. Regardless of WW or NW, the number of OTUs in the rhizosphere soil was the highest, followed by the heartwood. The number of OTUs in the sapwood was the smallest (Supplementary Table S1). The Wilcoxon rank-sum test was used to analyze the differences between the alpha-diversity indices of WW and NW, which showed that the Pielou’s evenness index, Shannon index, and Simpson index of WW-H were significantly lower than those of NW-H (Figures 5B–D). No significant differences in the richness, evenness, or diversity indices were detected in sapwood, root, and rhizosphere samples between WW and NW (Figure 5).

**Figure 6:**
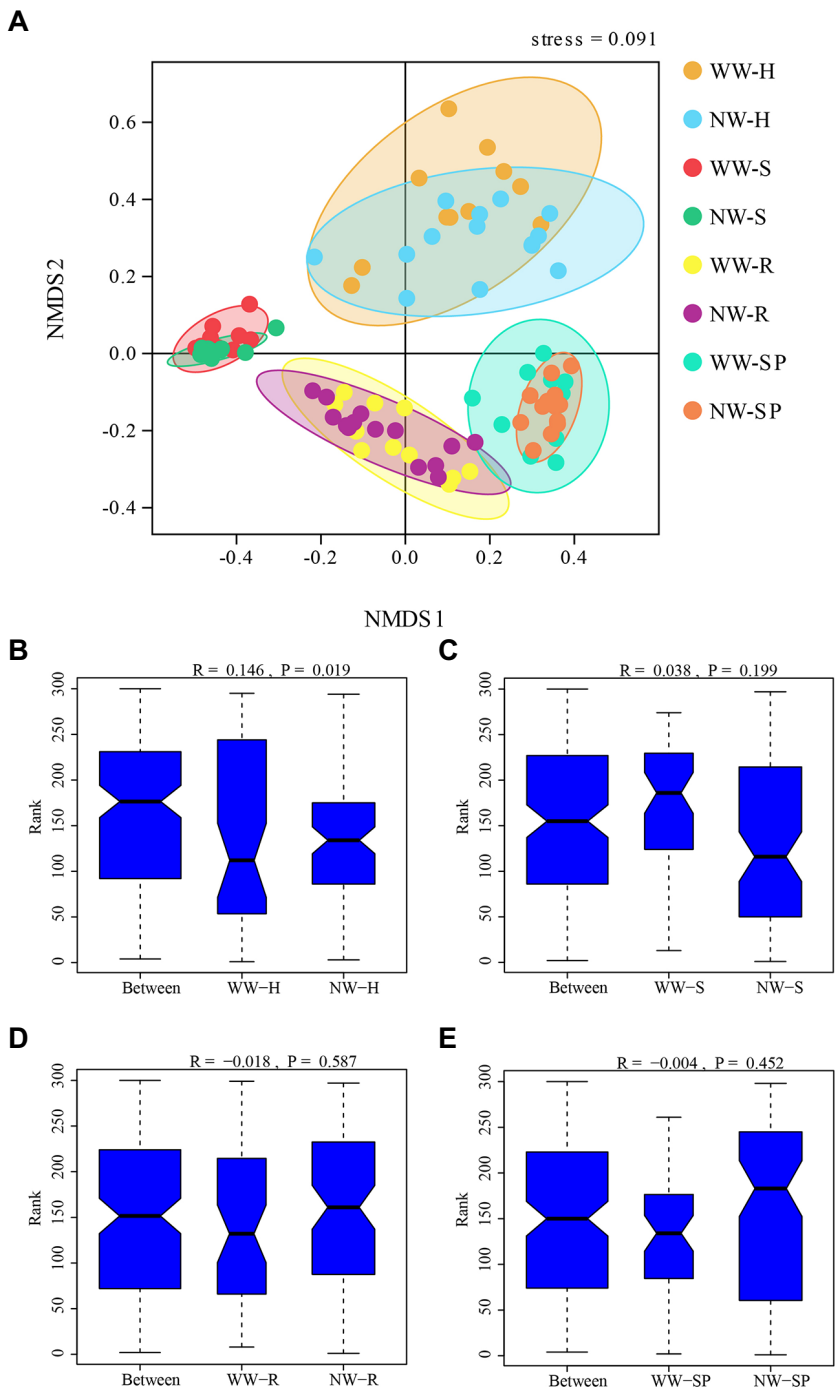
Beta diversity of the bacterial communities in each sample. **(A)** Non-Metric Multidimensional Scaling (NMDS) analysis of the bacterial community structure based on Bray–Curtis dissimilarity at the OTU level; **(B)** ANOSIM analysis of WW-H and NW-H; **(C)** ANOSIM analysis of the sapwood from wetwood trees (WW-S) and the sapwood from healthy trees (NW-S); **(D)** ANOSIM analysis of root tissue from wetwood trees (WW-R) and root tissue from healthy trees (NW-R); and **(E)** ANOSIM analysis of rhizosphere soil from wetwood trees (WW-SP) and rhizosphere soil from healthy trees (NW-SP).

To further compare the bacterial community structure between the WW and NW samples, a NMDS analysis combined with ANOSIM was performed (Figure 6). The results showed that bacterial community structure differed between healthy and diseased trees in the heartwood only, while sapwood, root, and rhizosphere soil bacterial communities were similar.

### Taxonomic Composition of the Bacterial Communities

**Figure 7:**
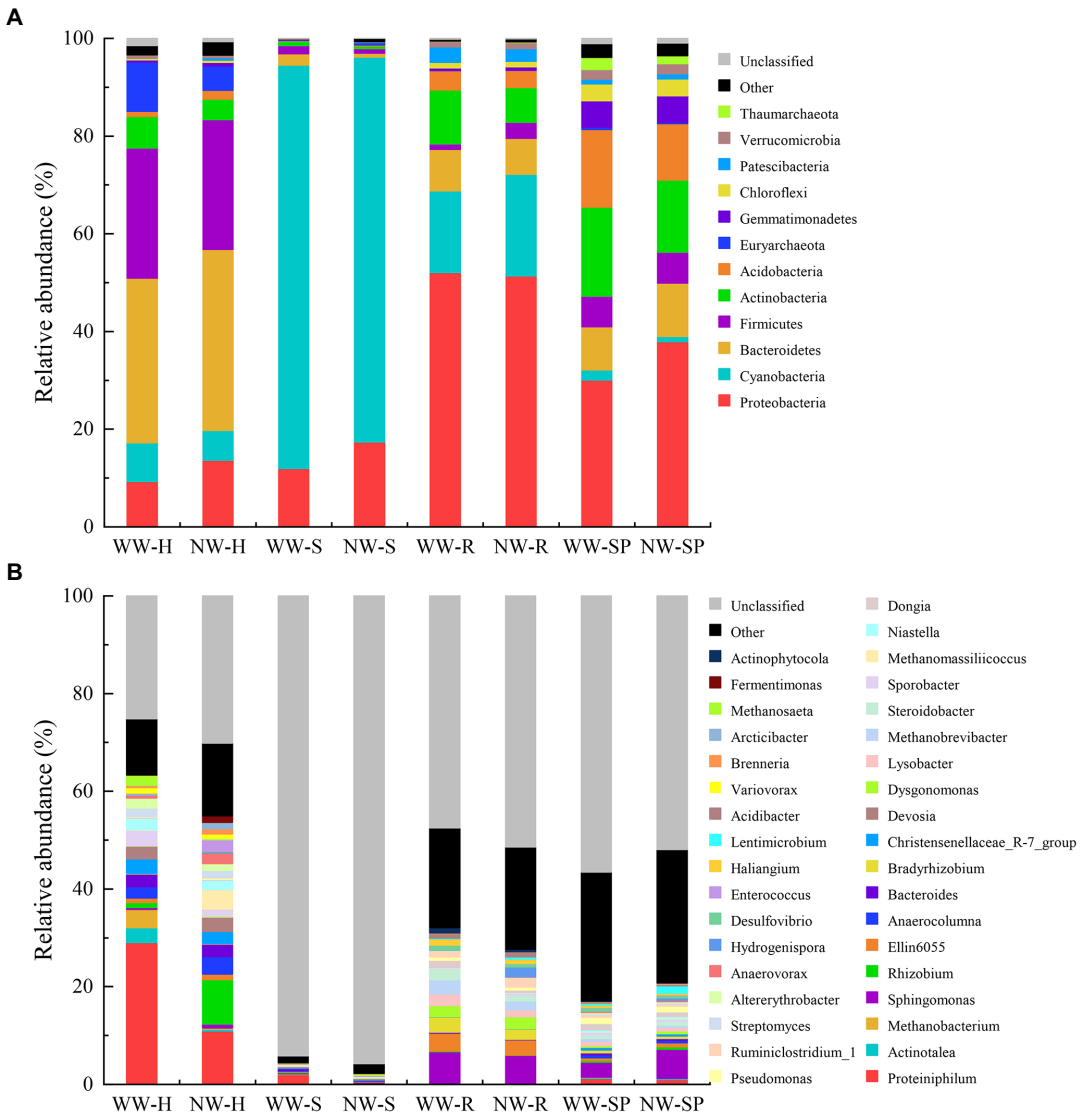
Relative abundance of species in the samples. **(A)** Relative abundance of bacterial taxa at the phylum level; **(B)** Relative abundance of bacterial taxa at the genus level. The figure shows species with relative abundance >1% of each group at each taxonomic level. WW-H, NW-H, WW-S, NW-S, WW-R, NW-R, WW-SP, and NW-SP represent heartwood of diseased trees, heartwood of healthy trees, sapwood of diseased trees, sapwood of healthy trees, root of diseased trees, root of healthy trees, rhizosphere soil of diseased trees, and rhizosphere soil of healthy trees, respectively.

A total of 41 phyla were identified from all samples. Bacteroidetes, Firmicutes, Euryarchaeota, Proteobacteria, and Cyanobacteria were the dominant bacterial phyla in the heartwood samples. The same was true for sapwood (excluding Euryarchaeota) and root tissue (excluding Euryarchaeota but including Actinobacteria). The main phyla were Proteobacteria, Actinobacteria, Acidobacteria, and Bacteroidetes in rhizosphere soil samples (Figure 7A).

Bacterial abundance at the genus level for all samples was shown in Figure 7B for breakdown. *Proteiniphilum* (29.01%), *Actinotalea* (3.70%), and *Methanobacterium* (3.08%) were the dominant genus in WW-H. *Proteiniphilum* (10.91%), *Dysgonomonas* (9.10%), and *Bacteroides* (4.05%) were the dominant genus in NW-H. *Proteiniphilum* (1.97%), *Anaerocolumna* (0.47%), and *Sphingomonas* (0.21%) were the dominant genus in WW-S. *Methanobrevibacter* (0.47%), *Christensenellaceae_R-7_group* (0.47%), and *Sphingomonas* (0.47%) were the dominant genus in NW-S. *Sphingomonas* was the dominant genre in root and rhizosphere soil of diseased and healthy trees.

Significant differences in the relative abundance of bacterial phyla and genera between the samples were revealed according to a Wilcoxon rank-sum test. There were significant differences in the abundances of a few number of phyla between diseased and healthy trees (*p* < 0.05, Supplementary Table S2). There were 73 genus had significant differences between WW-H and NW-H (*p* < 0.05, Supplementary Table S3). For example, the relative abundances of *Proteiniphilum*, *Actinotalea*, and *Methanobacterium* were significantly higher in WW-H than NW-H. For samples from sapwood, root and rhizosphere soil, we observed significant differences of 41, 25, and 29 genus between diseased and healthy trees, respectively.

### Correlation Between Endophytic Bacteria and the Chroma Value, Wet Heartwood Rate, and the Wood Properties

**Figure 8:**
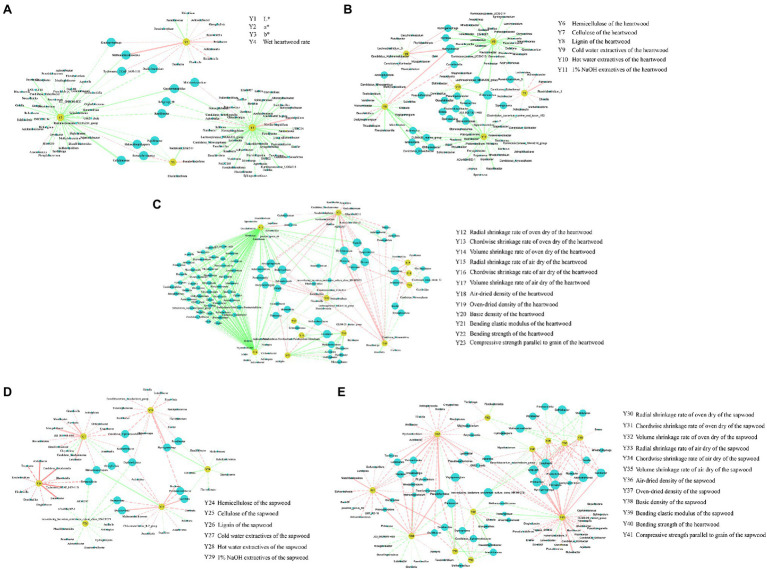
Spearman correlation network diagram of the endophytic bacteria and the chroma value, wet heartwood rate, and the wood properties. **(A)** Spearman correlation between the heartwood endophytic bacteria and the chroma value and wet heartwood rate; **(B)** Spearman correlation between heartwood endophytic bacteria and the chemical properties of the heartwood; **(C)** Spearman correlation between the heartwood endophytic bacteria and the physical and mechanical properties of the heartwood; **(D)** Spearman correlation between the sapwood endophytic bacteria and the chemical properties of sapwood; **(E)** Spearman correlation between sapwood endophytic bacteria and the physical and mechanical properties of sapwood. The blue nodes represent the bacterial genus, and the yellow nodes represent the wood property indicators. The size of the blue node represents the number of connections to that node. The solid line represents highly significant, and the dashed line represents significant. The red line represents a positive correlation, and the green line represents a negative correlation. The thickness of the connecting line represents the strength of the correlation.

We performed Spearman correlation analysis to study the relationship between endophytic bacteria and the chroma value, wet heartwood rate, and wood properties. The results showed that most of the heartwood endophytic bacteria were negatively correlated with the chroma value, wet heartwood rate, and heartwood wood properties. *Proteiniphilum* was highly significantly positively correlated with wet heartwood rate, significantly negatively correlated with compressive strength parallel to the grain of the heartwood, and highly significantly negatively correlated with the bending elastic modulus of the heartwood. *Methanobacterium* was significantly positively correlated with the wet heartwood rate, significantly negatively correlated with a^*^ and oven-dried radial shrinkage of the heartwood, and highly significantly negatively correlated with oven-dried volume shrinkage and air-dried radial shrinkage of the heartwood (Figures 8A–C; Supplementary Table S4).

*Propionivibrio*, *Candidatus_Xiphinematobacter*, *Desulfopila*, and *Thiobacillus*. *Proteiniphilum* was significantly negatively correlated with oven-dried radial shrinkage of the sapwood, and highly significantly negatively correlated with compressive strength parallel to the grain of the sapwood. *Methanobacterium* was significantly negatively correlated with oven-dried volume shrinkage and air-dried chordwise shrinkage of the sapwood. *Methanoculleus* was significantly negatively correlated with air-dried radial shrinkage and air-dried volume shrinkage of the sapwood (Figures 8D,E; Supplementary Table S5).

**Figure 9:**
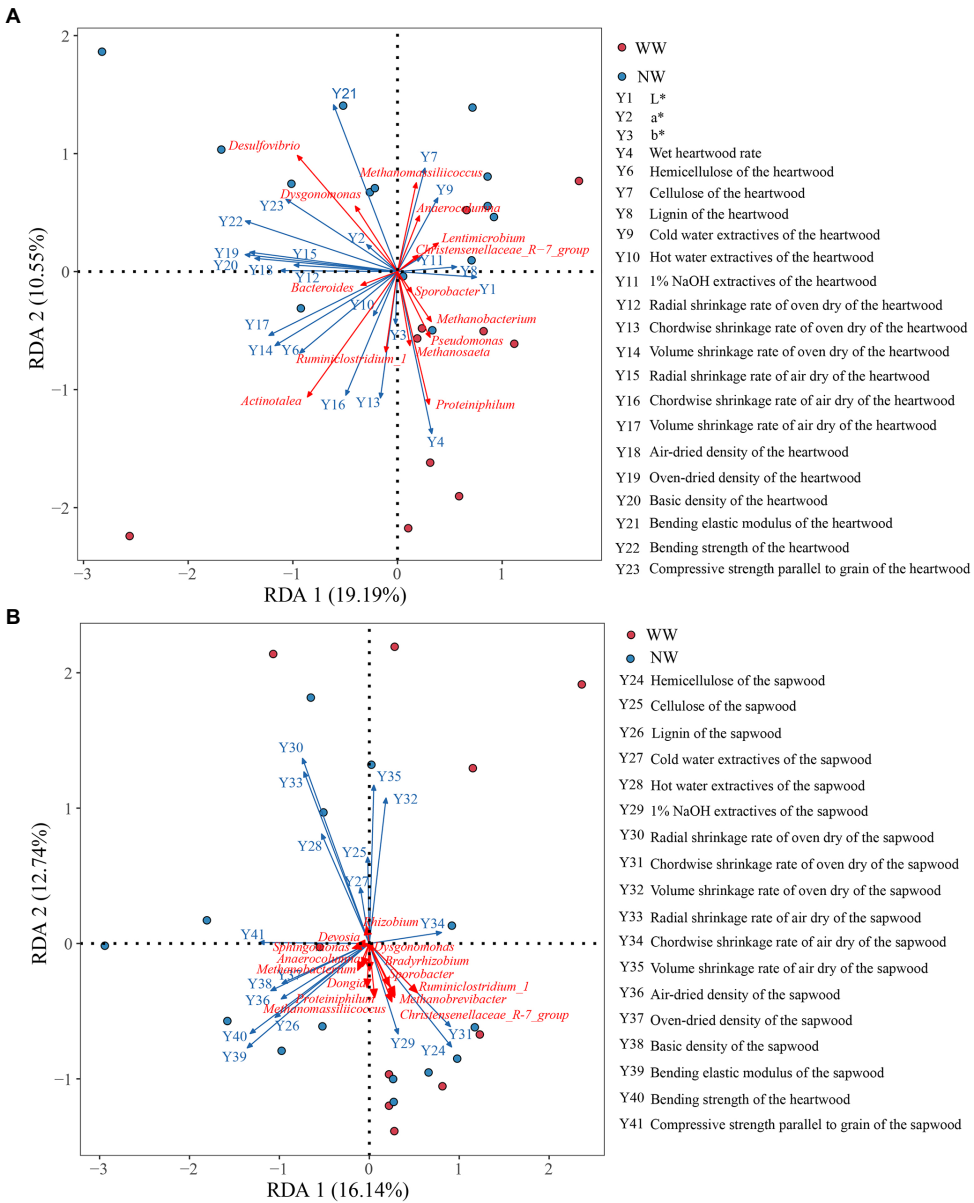
db-RDA analysis of the endophytic bacteria and the chroma value, wet heartwood rate, and the wood properties. **(A)** The relationship between endophytic bacteria of the heartwood and wood variables of the heartwood; **(B)** The relationship between endophytic bacteria of the sapwood and wood variables of the sapwood. WW and NW represent diseased and healthy trees, respectively.

The db-RDA analysis was performed to identify the most important taxa liked to the wood variables (Figure 9). The main endophytic bacteria affecting the wood variables of the heartwood were *Desulfovibrio*, *Actinotalea*, *Proteiniphilum* and *Methanomassiliicoccus*. Additionally, *Proteiniphilum*, *Actinotalea*, *Methanosaeta*, and *Methanobacterium* were positively correlated with wet heartwood rate, but they were negatively correlated with cellulose of the heartwood (Figure 9A). In comparison, endophytic bacteria of the sapwood had little effect on wood variables of sapwood (Figure 9B).

## Discussion

In this study, we showed that *Populus* × *euramericana* cv. “74/76” presents typical characteristics of the wet heartwood disease, such as dark heartwood color and a foul odor of fatty acids. The chromophore and auxochrome in the wood structure are connected with an aromatic ring structure to form a conjugated system, which absorbs and reflects visible light to make the wood reflect different colors. These groups are mainly present in lignin and the extractives (Yao et al., 2012). Most heartwood extractives include compounds, such as polyphenols, resins, organic acid salts, and fats, which also cause a darkening of the wet heartwood (Zhu Ge et al., 1997; Burtin et al., 2000).

Most studies have used traditional culture methods to isolate and purify the dominant bacterial strains from the heartwood tissue of wetwood (Alizadeh et al., 2017a; Song et al., 2018; Basavand et al., 2021), or to study the microorganisms by comparing the microbial communities of the wetwood and normal sapwood tissues of diseased trees (Streichan, 1986; Kovaleva et al., 2015). In this study, the high-throughput sequencing method was used to identify and compare the bacterial microbial diversity of heartwood, sapwood, root tissue, and rhizosphere soil of diseased and healthy trees. The bacterial evenness and diversity indices in the heartwood of diseased trees were significantly lower than that of healthy trees. However, the alpha-diversity indices of sapwood, root, and rhizosphere soil between diseased and healthy trees were no significant differences. These results suggest that wetwood affected the diversity and evenness of the endophytic bacterial community in the heartwood, but had no effect on the richness, evenness, and diversity of endophytic bacteria in the sapwood, endophytic bacteria of the root tissue, or rhizosphere soil bacteria. Endophytic bacteria, make-up part of plants microbiome, these microorganisms are associated to every single plant organ, where they are protected from environmental changes and therefore mostly might be affected by changes occurring within plant tissues (Ryan et al., 2007). The higher diversity of endophytic bacteria in heartwood of healthy trees could be an indication of pathogen suppression which delayed or prevented the diseased expression. Suhaimi et al. (2017) have also reported that the higher diversity of endophytic bacteria in the non-symptomatic bacterial wilt-diseased banana plants compared to the symptomatic plant.

A NMDS analysis revealed a significant difference in the bacterial community structure of heartwood between the diseased and healthy trees, indicating that the functions of the two microenvironments differ. And the bacterial community structure of sapwood, root tissue, and rhizosphere soil samples were not significantly different between diseased and healthy trees, indicating that wet heartwood disease could only cause changes in the endophytic bacterial community in the heartwood, which may be one of the characteristics of trees suffering from wet heartwood disease.

A db-RDA found that the wet heartwood rate was positively correlated with the relative abundance of *Proteiniphilum*, *Actinotalea*, and *Methanobacterium*. The cellulose content of diseased trees’ heartwood was lower than that of healthy trees’ heartwood, and cellulose content was negatively correlated with the relative abundances of *Proteiniphilum*, *Actinotalea*, and *Methanobacterium*. This may be due to the small sample size or the uneven distribution of these three bacteria in samples. The relative abundances of *Proteiniphilum*, *Actinotalea*, and *Methanobacterium* in diseased trees’ heartwood were significantly higher than in healthy trees’ heartwood, particularly *Proteiniphilum*, which was significantly enriched in diseased trees’ heartwood. According to reports, the main gases in wet heartwood are CH_4_, N_2_, and CO_2_, with a small amount of H_2_ but no O_2_ (Schink et al., 1981a; Schink and Ward, 1984). *Proteiniphilum* is a facultative anaerobe and potent cellulolytic bacterium that directly degrades lignocellulose to CO_2_, formate, and acetate via aerobic respiration and anaerobic fermentation (Hahnke et al., 2016; Wu et al., 2020). *Actinotalea* is an anaerobic bacterium from Cellulomonadaceae family. Cellulomonadaceae family was known as the functional bacteria to degrade cellulose, and the intermediate metabolites could be used as carbon source for denitrification (Rusznyak et al., 2011; Feng et al., 2017). Wetwood is an environment in which anaerobic cellulolytic bacteria might be expected to thrive. And Warshaw et al. (1985) isolated obligately anaerobic, mesophilic, cellulolytic bacteria from the wetwood of elm and maple trees. *Methanobacterium* is a typical hydrogen-oxygen methanogen that produces CH_4_ from formate and H_2_/CO_2_ and is often isolated from wetwood (Zeikus and Ward, 1974; Yu et al., 2019; Zhang et al., 2019). In addition to *Methanobacterium*, the relative abundances of *Methanobrevibacter*, *Methanosaeta*, and *Methanoculleus* of diseased trees’ heartwood were also greater than those of healthy trees’ heartwood. These bacteria belong to Euryarchaeota, which converts acetate, H_2_ and CO_2_ to CH_4_ by acetic acid and hydrogenotrophic methanogenesis, respectively (Wagner et al., 2013; Oszust et al., 2018; Lee and Hwang, 2019). Based on the function of these bacteria, this study speculated that anaerobic bacteria with specific functions such as *Proteiniphilum* and *Actinotalea* hydrolyzed cellulose and easily degradable carbohydrates in wood into sugars, amino acids, and long-chain fatty acids. These products provided methanogens with the substrates they need to grow and convert methane. However, these speculations need further verification.

In this study, wetwood disease caused a shift in the heartwood bacterial community of *Populus* × *euramericana* cv. “74/76,” but the disease had little effect on the bacterial communities of sapwood, root tissue, or rhizosphere soil. Song et al. studied the infection route of the pathogenic bacteria causing poplar wetwood disease and found that the stem wound infection was the main pathway for pathogenic bacteria to infect poplars, and neither leaf cuttings nor root irrigation infected the poplars (Song et al., 2017). Therefore, carrying out poplar infection test can further explore the pathogenic mechanisms of poplar wetwood disease. In addition, the use of the antagonism between microorganisms to screen the bacteria antagonistic to the pathogenic bacteria of wet heartwood would be of great significance for the biological control of poplar wetwood disease.

## Data Availability Statement

The datasets presented in the study are deposited in the National Center for Biotechnology Information (NCBI) repository, accession number PRJNA832960.

## Author Contributions

JF conceived the study, analyzed the data, and edited the manuscript. SW and CD collected and analyzed the data. CM and XC analyzed the data. JW, MY, and XS designed the experiments and revised the manuscript. All authors contributed to the article and approved the submitted version.

## Funding

This research was funded by the National Key Research and Development Program of China (grant number 2021YFD2201200) and Province Key Research and Development Program of HeBei (grant number 21326301D).

## Conflict of Interest

The authors declare that the research was conducted in the absence of any commercial or financial relationships that could be construed as a potential conflict of interest.

## Publisher’s Note

All claims expressed in this article are solely those of the authors and do not necessarily represent those of their affiliated organizations, or those of the publisher, the editors and the reviewers. Any product that may be evaluated in this article, or claim that may be made by its manufacturer, is not guaranteed or endorsed by the publisher.

## Supplementary Material

The Supplementary Material for this article can be found online at: https://www.frontiersin.org/articles/10.3389/fmicb. 2022.868078/full#supplementary-material
