# Supplementary figures and images for: Correlation Analysis of the Bacterial Community and Wood Properties of Populus × euramericana cv. “74/76” Wet Heartwood
Source: Front Microbiol. 2022 Jul 4;13:868078. doi: 10.3389/fmicb.2022.868078 (PMC9289670; doi:10.3389/fmicb.2022.868078)

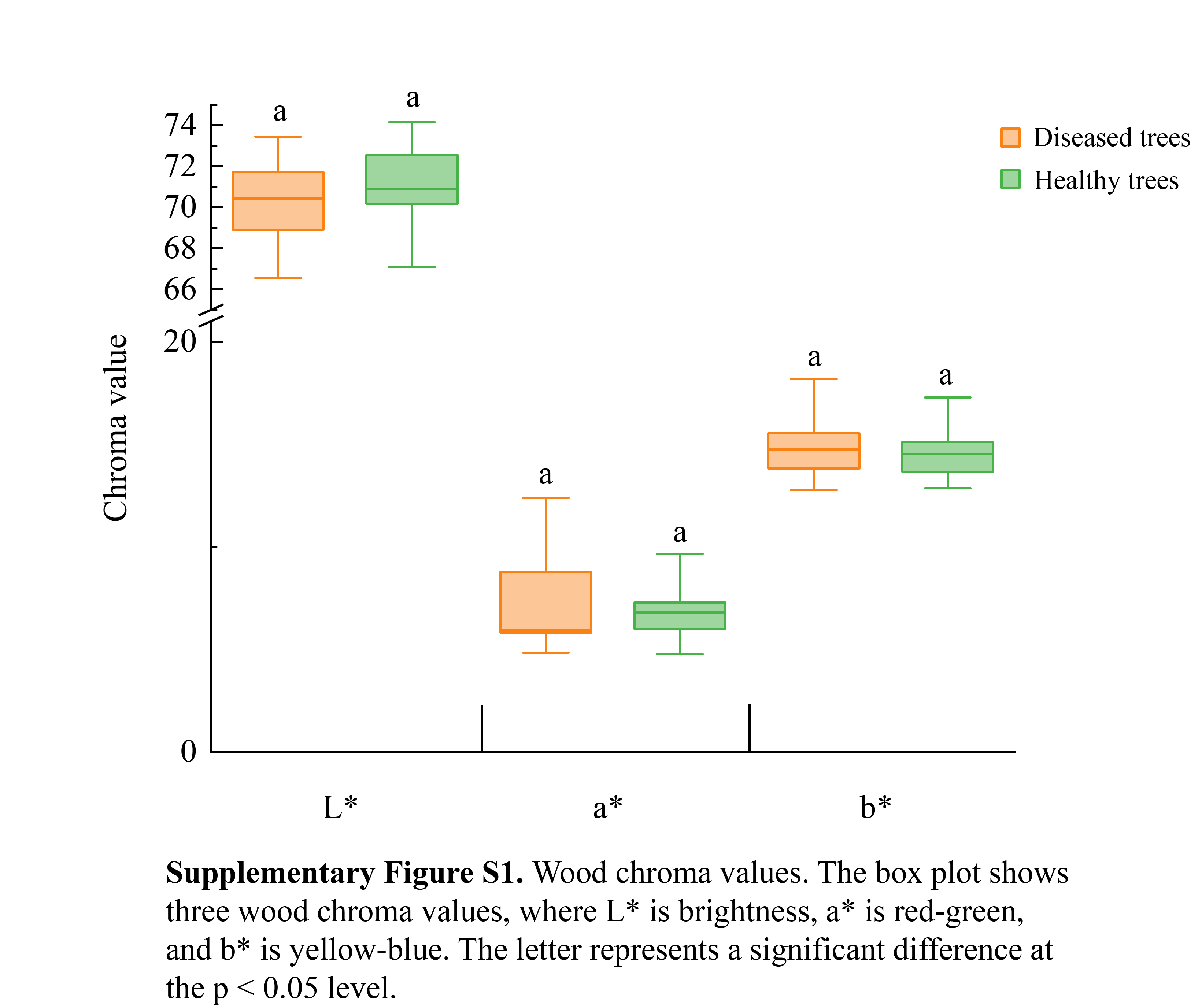

Supplement: Supplementary file 6 [file Image_1.JPEG]

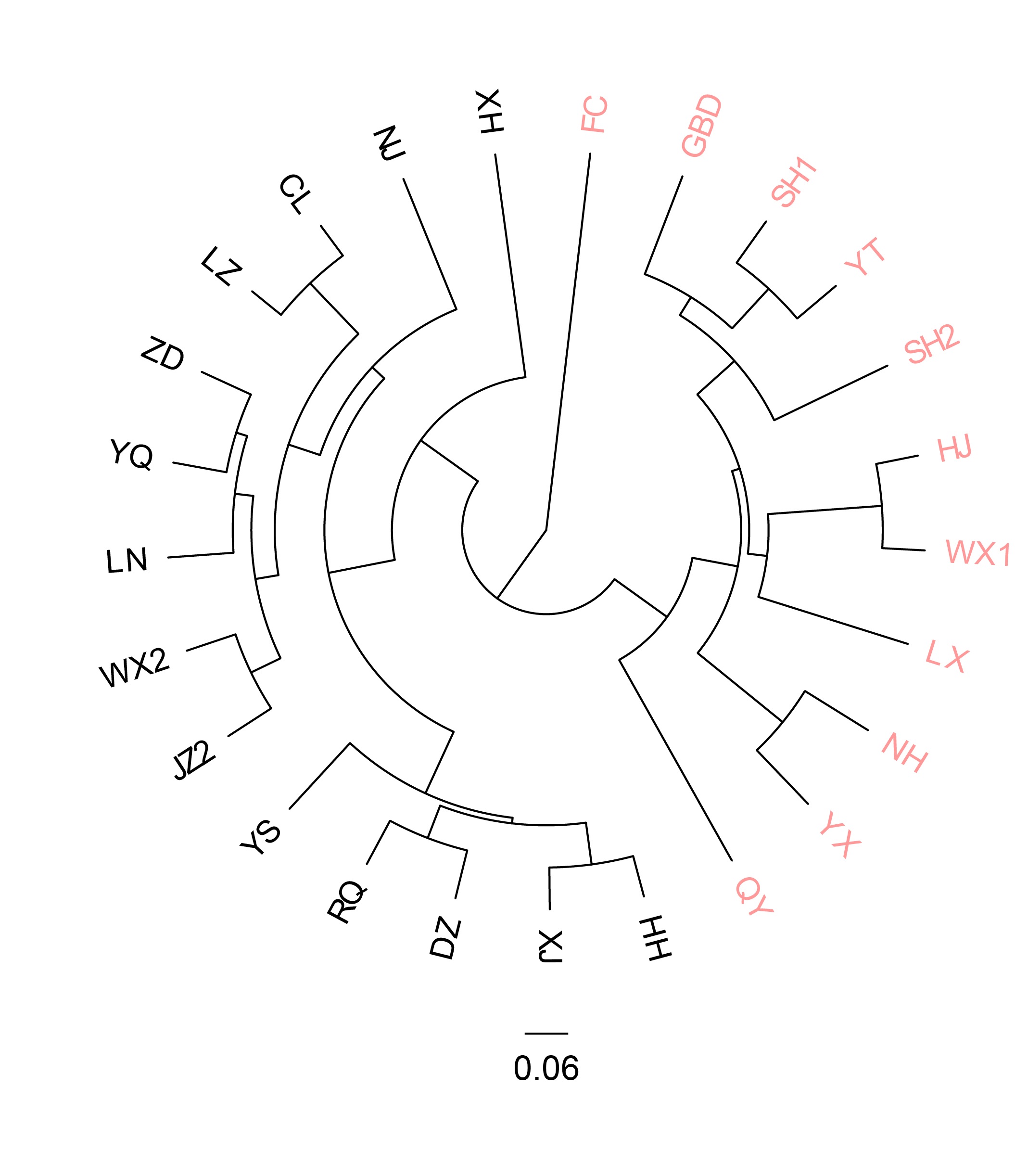

Supplement: Supplementary file 7 [file Image_2.JPEG]
